# Mr. Charles Frederick Long

**Published:** 1910-08-06

**Authors:** 


### OBITUARY.

The death is announced of Mr. Charles Frederick Long, M.D., of Ottery St. Mary, late of Canterbury, aged 72.

